# Prediction of Grain Size in a High Cobalt Nickel-Based Superalloy

**DOI:** 10.3390/ma16175776

**Published:** 2023-08-23

**Authors:** Jingzhe Wang, Siyu Zhang, Liang Jiang, Shesh Srivatsa, Zaiwang Huang

**Affiliations:** 1 State Key Laboratory of Powder Metallurgy, Central South University, Changsha 410083, China; wangjingzhe@csu.edu.cn (J.W.);; 2 Powder Metallurgy Research Institute, Central South University, Changsha 410083, China; 3 Institute for Advanced Studies in Precision Materials, Yantai University, Yantai 264005, China; 4 Srivatsa Consulting LLC, Cincinnati, OH 45249, USA

**Keywords:** high-temperature alloys, powder metallurgy, crystal structure, computer simulations, compression test, flow stress

## Abstract

With the advancement in computational approaches and experimental, simulation, and modeling tools in recent decades, a trial-and-validation method is attracting more attention in the materials community. The development of powder metallurgy Ni-based superalloys is a vivid example that relies on simulation and experiments to produce desired microstructure and properties in a tightly controlled manner. In this research, we show an integrated approach to predicting the grain size of industrial forgings starting from lab-scale cylindrical compression by employing modeling and experimental validation. (a) Cylindrical compression tests to obtain accurate flow stress data and the hot working processing window; (b) double-cone tests of laboratory scale validation; (c) sub-scale forgings for further validation under production conditions; and (d) application and validation on full-scale industrial forgings. The procedure uses modeling and simulation to predict metal flow, strain, strain rate, temperature, and the resulting grain size as a function of thermo-mechanical processing conditions. The models are calibrated with experimental data until the accuracy of the modeling predictions is at an acceptable level, which is defined as the accuracy at which the results can be used to design and evaluate industrial forgings.

## 1. Introduction

Powder metallurgy nickel-based superalloys are widely used to fabricate high-pressure turbine disks in aircraft due to their excellent performance at elevated temperatures. In past decades, the alloy compositions have always been optimized to meet higher operating temperatures. Controlling dynamic recrystallization grain size in a repeatable manner is a major concern during the hot working of structural materials, especially for large-scale components, since it significantly affects subsequent processing [1,2], heat treatment [3,4], and properties [5,6]. There is a need to accurately predict the dynamic recrystallization grain size, which depends on deformation temperature, strain, and strain rate [7,8]. To realize this, two concerns must be addressed: (1) precisely predicting local processing parameters throughout the component; (2) having precise dynamic recrystallization grain size models based on simulated local parameters and using experimental results to calibrate their accuracy. To date, an integrated approach that can be used to predict grain size using a step-wise method from lab-scale specimens to full-scale forgings is lacking.

Flow stress was often obtained using cylindrical compression specimens over a range of temperatures, strains, and strain rates. From the measured load-stroke curves, the corresponding true stress true strain data [9,10] (herein denoted by flow stress) were obtained [11]. The flow stress was then corrected by considering the effects of friction [12,13], adiabatic temperature rise, and non-homogeneous deformation [14,15]. The flow stress data were validated by conducting double-cone (DC) tests to generate a range of strain and strain rates for a single specimen [16,17]. The measured load-stroke and final dimensions from the DC tests were compared with finite element models using the corrected flow stress data. When the difference between the predicted and experimental load-stroke data as well as the final specimen dimension was within an acceptable range, typically less than 5%, the flow stress and deformation models were believed to be accurate enough and could be used to simulate sub-scale and full-scale industrial forgings. The predicted strain, strain rate, and temperature can also be used in dynamic recrystallization grain size models. This procedure ensures that each step is validated before using the data in the subsequent step. If an attempt is made to directly validate the dynamic recrystallization grain size models, it is not difficult to isolate the cause of any discrepancy between the predicted and measured dynamic recrystallization grain sizes.

Certain positions on the deformed double cones were selected for experimental measurements and used to validate the simulated dynamic recrystallization grain size [18]. The positions were selected to cover a range of thermo-mechanical conditions that are typically encountered with industrial forgings [19]. When the difference between the experimentally observed and the simulated dynamic recrystallization grain size falls to an acceptable value, the dynamic recrystallization grain size model is considered validated and suitable for prediction at any position within the forging. In this research, we will provide an integrated approach to predicting dynamic recrystallization grain size using a step-wise method from laboratory specimens to full-scale industrial forgings.

## 2. Materials and Methods

A high-cobalt powder metallurgy Ni-based superalloy was designed by our research group [20,21]. The alloy powder was prepared by argon gas atomization and sieved using a mesh size of 10–63 µm. The alloy powder was consolidated using hot isostatic pressing at 1100 °C/150 MPa for 4 h. Subsequently, hot extrusion was performed using an extrusion ratio of 6:1 at 1100 °C. To reduce the retained metallurgical strain, the extruded billet was heated at 1080 °C for 2 h and then air cooled. The cylindrical and double-cone specimens were machined from the extruded billets using electrical discharge machining. 

**Figure 1:**
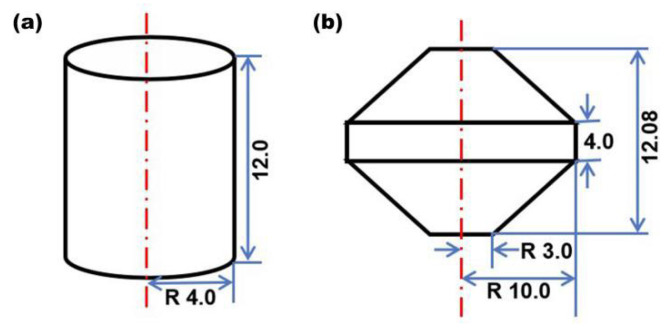
The geometry of cylinder (**a**) and double-cone specimens (**b**).

**Figure 2:**
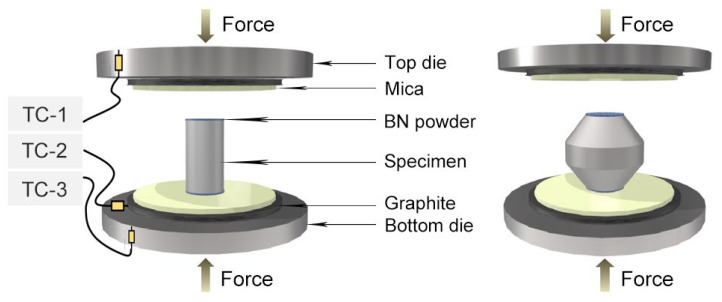
Schematic of cylindrical and double-cone specimens for hot compression. Three-layered structures composed of boron nitride powder, mica, and graphite foil are coated on the bottom and top planes of specimens.

Cylindrical specimens (Φ8 × 12 mm height) and double-cone (DC) specimens (Φ20 × 16 mm height) shown in Figure 1 were used for hot compression experiments. Three layers composed of boron nitride powder, mica, and graphite foil (as shown in Figure 2) were applied on the specimen’s bottom and top ends to minimize interfacial friction between the dies and the specimen. Prior to hot compression, the furnace was heated to the test temperature, and then the specimen was loaded into the furnace until it reached the test temperature. The specimen was soaked for 15 min to obtain a uniform temperature.

Compression tests were conducted over a range of temperatures from 1010 °C to 1121 °C. Three strain rates (0.0032 s^−1^, 0.01 s^−1^, and 0.032 s^−1^) were chosen for compression, and the height reduction of the specimens was set to 50%, corresponding to a true effective compression strain of 69.3%. After compression, the specimens were immediately pulled out and quenched in the water to freeze the microstructure.

The compressed specimens were cut into two halves along the center-axis plane (along the compression direction) and prepared by mechanical grinding, polishing, and vibration polishing for electron backscatter diffraction (EBSD) observation (detector: Oxford instrument, software: AztecCrystal, data analysis software, HKL Channel 5). Under a scanning electron microscope (SEM), the acceleration voltage was 15 kV and the working current was 3 mA. Finite element modeling (FEM) was performed using DEFORM-2D software (Scientific Forming Technology Cooperation, Columbus, OH, USA).

## 3. Results and Discussion

**Figure 3:**
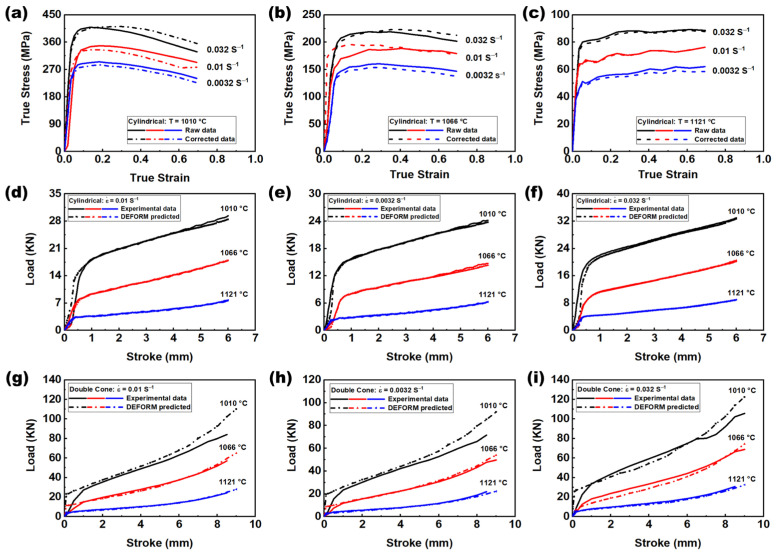
Correction of flow stress data. (**a**–**c**) True stress-true strain curves were obtained from load-stroke data after two-step correction. (**d**–**f**) Comparison of the measured load-stroke with that predicted by the corrected flow stress. (**g**–**i**) Prediction of load-stroke curves of double-cone specimens using corrected flow stress of cylindrical compression over a range of strain rate and temperature.

Figure 3 shows cylindrical compression true stress true strain curves (Figure 3a–c) over a range of temperatures and strain rates for the same nominal compressive strain of 50%. The flow stress is lower at higher temperatures and lower strain rates. As has been widely demonstrated in previous research, alloys compressed at a higher temperature and lower strain rate have lower strength [9,22]. The true stress true strain curves were obtained from the load-stroke curves after corrections due to interfacial friction and adiabatic temperature rise (the details of flow curve correction can be found in our previous work [21]). The load-stroke predicted by the corrected flow curve is shown in Figure 3d–f, which is in good agreement with the experimentally measured ones.

The corrected flow curve data were validated using DC compression data. Compression tests for lab-scale DC specimens can produce strains and strain gradients similar to full-scale forgings. Figure 3g–i show load-stroke data at different temperatures and strain rates. The predicted load-stroke data are very close to the experimental results. Hence, the simulated working parameters in the deformed DC specimens can be used with confidence for microstructure models. If the agreement with the predicted and measured loads is not at an acceptable level, the flow stress must be corrected again.

**Figure 4:**
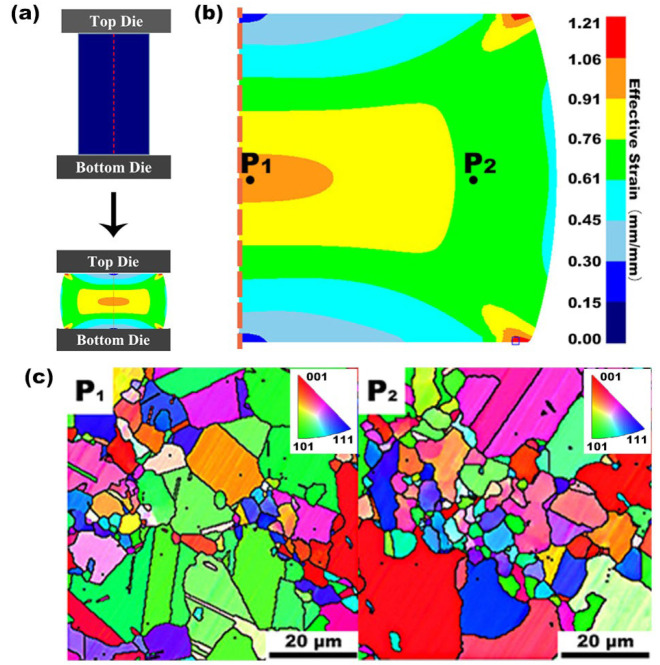
Dependence of grain size on the position inside a deformed cylindrical specimen. (**a**) Geometrical change of cylindrical specimen and effective strain distribution after 50% compression strain at 1121 °C and 0.01 s^−1^. (**b**) Effective strain varies from the rim to the core regions based on FEM. (**c**) EBSD measurement of grain size at two typical positions on a different effective stain.

The calibration and validation of the constitutive model of the alloy and the finite-element hot deformation simulation are critical to predicting the location-specific process conditions within a laboratory specimen or a forging component. Thus, the accurate simulation of local deformation parameters is the primary aim since these are necessary to calculate microstructure information such as grain size and dynamic recrystallization (DRX) fraction [13]. Figure 4a shows geometric changes in the compression of a cylindrical specimen. The FEM results in Figure 4b show that there are strain gradients in the deformed specimen. Figure 4c shows the microstructure of the cylindrical specimen at positions P_1_ (hub area) and P_2_ (effective strain corresponds to 0.7), which are dependent on local working parameters. Based on our simulation, the strain gradient between P_1_ and P_2_ is relatively small, corresponding to a small change in grain microstructures.

**Figure 5:**
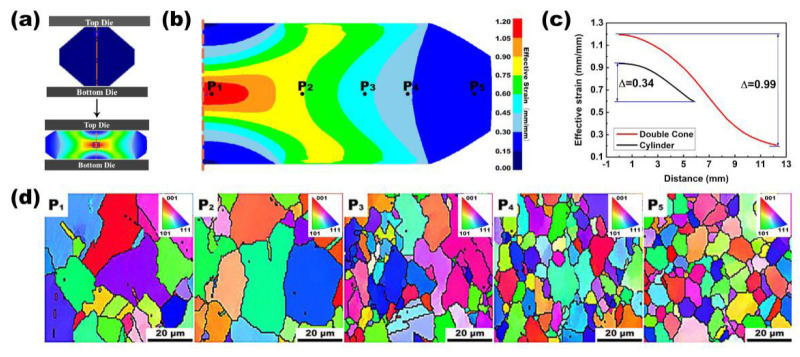
Dependence of grain size on the position inside a deformed double-cone specimen. (**a**) The geometry of the double-cone specimen and effective strain distribution after 50% compression strain at 1121 °C and 0.01 s^−1^. (**b**) Effective strain varies from the rim to the core regions based on FEM. (**c**) Comparison of the variation range of centerline equivalent strain between cylindrical and DC specimens. (**d**) EBSD measurement of the DC specimen’s grain size at five typical positions with different effective strains.

As mentioned previously, accurate simulation of local deformation parameters is the prime aim of calculating microstructure information such as grain size and dynamic recrystallization fraction. Figure 5a shows geometric changes in the compression of the DC specimen. The finite element simulation (FES) results in Figure 5b show that there is a strain gradient from the hub to the rim of a compressed DC specimen at 1121 °C and 0.01 s^−1^. Figure 5c shows the effective strain variation curves of the center lines of the cylindrical and DC specimens. The central effective strain range of the DC specimen (0.21–1.2) is significantly larger than that of the cylindrical specimen (0.59–0.93). The change in the effective strain is small from the hub to the rim of the cylindrical specimen, and the change in the corresponding microstructure, such as grain size, is also small (Figure 5c,d). In contrast, multiple positions span a wide effective strain range from the hub to the rim of the deformed DC specimen. Five different locations (P_1_–P_5_ assigned in Figure 5b) were selected, and the grain size and DRX fraction were measured using the EBSD method. Microscopic observations (Figure 5d) show that grain sizes are strongly dependent on position (local processing parameters).

Moreover, we can compare grain size information between cylindrical and DC specimens. The grain size information from cylindrical and DC specimens was compared. P_1_ and P_2_ in Figure 4c correspond to P_2_ and P_3_ in Figure 5d. The grain morphology and grain size of cylindrical and DC specimens are similar at the same effective strain position. With the increase in effective strain at the same hot compression temperature, the grain morphology evolves from equiaxed grain to non-equiaxed grain with an irregular shape, and many fine grains appear in the middle part. The appearance of these fine grains indicates the onset of recrystallization nucleation in the specimen.

The Avrami equations [23,24] (Equations (1)–(3)) are used to calculate the DRX volume fraction [25,26] and its average grain size [27], where A_1_, n_1_, A_2_, n_2_, A_3_, and n_3_ are the material constants, Q_1_ and Q_2_ are the material activation energies, $\dot{\varepsilon }$ (s^−1^) is the strain rate, ε is the true strain, ε_c_ is the critical strain, ε_0.5_ is the strain when the DRX volume fraction is 50%, R is the gas constant (8.314 J/(mol•K)), and T (°C) is the absolute temperature.

(1) $$\varepsilon_{0.5}=A_{1}{\dot{\varepsilon }}^{n_{1}}\exp\left(\frac{Q_{1}}{\mathrm{RT}}\right)$$

(2) $$X_{\mathrm{DRX}}=1-\exp\left[A_{2}{\left(\frac{\varepsilon -\varepsilon_{c}}{\varepsilon_{0.5}}\right)}^{n_{2}}\right]$$

(3) $$d_{\mathrm{DRX}}=A_{3}{\dot{\varepsilon }}^{n_{3}}\exp\left(\frac{Q_{2}}{\mathrm{RT}}\right)$$

Based on the alloy composition used in this research, these equations are determined:

(4) $$\varepsilon_{0.5}=8.71\times 10^{-6}{\dot{\varepsilon }}^{-0.24}\exp\left(\frac{114071}{\mathrm{RT}}\right)\;$$

(5) $$X_{\mathrm{DRX}}=1-\exp\left[-0.604{\left(\frac{\varepsilon -\varepsilon_{c}}{\varepsilon_{0.5}}\right)}^{0.401}\right]$$

(6) $$d_{\mathrm{DRX}}=1.88\times 10^{6}{\dot{\varepsilon }}^{-0.17}\exp\left(\frac{167323}{\mathrm{RT}}\right)$$

**Figure 6:**
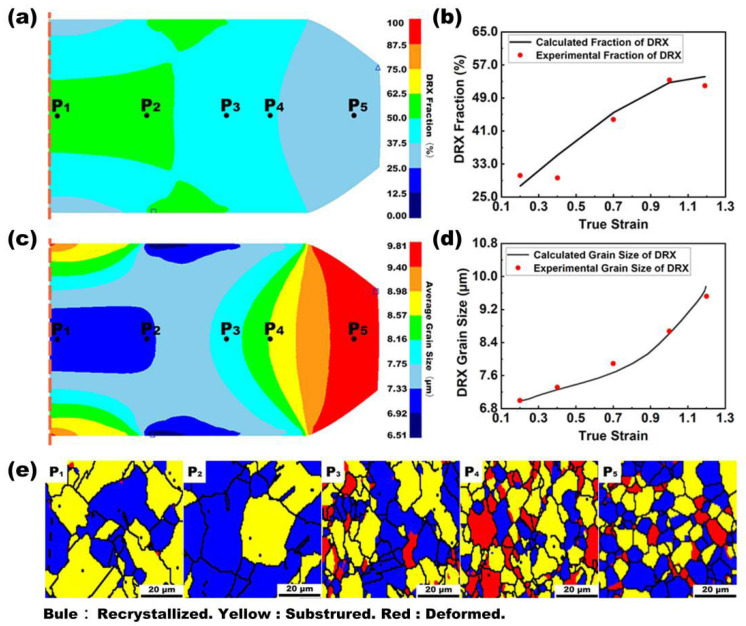
Experimental and simulated results of DRX inside a double-cone specimen after 50% compression strain at 1121 °C and 0.01 s^−1^. (**a**) The DRX volume fraction varies from the hub to the rim of the deformed specimen. (**b**) Comparison of experimental and calculated DRX volume fractions. (**c**) DRX grain size varies from the hub to the rim of the deformed specimen. (**d**) Comparison of experimental and calculated DRX grain sizes. (**e**) DRX distribution obtained by EBSD.

The equations were used as an input into FEM. To validate modeling results, experimental results were compared with the modeling results at five different positions (Figure 6a,c). The grain size can be determined based on EBSD measurements (Figure 6e). The difference should be less than 5% for the next step.

Figure 6a is a contour plot of dynamic recrystallization (DRX) volume fraction (DRVF) at different positions (Equations (4) and (5)). The DRVF decreases from the hub to the rim of the deformed DC specimen. A comparison of the experimental and predicted DRX volume fractions is shown in Figure 6b. Similarly, the average DRX grain size can be calculated using Equation (6), and the DRX grain size varies from the hub to the rim of the deformed specimen, as shown in Figure 6c. A comparison of the experimental and predicted results is shown in Figure 6d,e which show that the DRX, substructure, and deformed grains depend on local deformation parameters. The DRVF of the DC specimens increases with the increase in the true strain, but the slope rapidly decreases. In contrast, the grain size of the DRX shows similar behavior, but the slope increases with the increasing true strain. The prediction and experimental results of the DRX volume fraction and the grain size demonstrate the accuracy of the model and the feasibility of the method.

**Figure 7:**
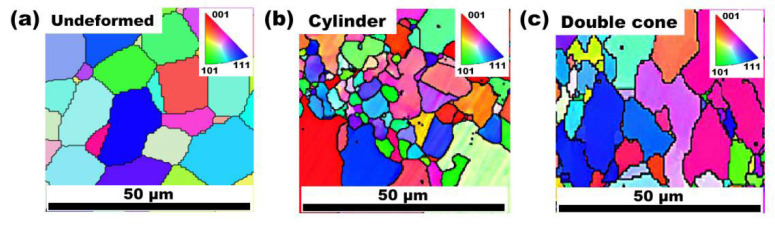
The microstructural characterization of the material before and after the hot compression tests by EBSD. (**a**) The initial microstructure of the material before the hot compression tests. (**b**) The microstructure of the cylindrical specimen after the hot compression tests. (**c**) The microstructural characteristics of the double-cone specimen after the hot compression tests.

Figure 7 shows the result of the microstructural characterization of the material before and after the hot compression tests by EBSD. The results show that the initial microstructure is equiaxed with uniform grain size before the hot compression test. After the hot compression test, the initial microstructure of the specimen is deformed, and uniform equiaxed crystals almost do not exist. In their place are newly formed, dynamically recrystallized small grains and morphologically deformed, irregular large grains. Hot compression not only causes the deformation of the original grain but also destroys the grain boundaries in the original material structure. The newly nucleated, dynamically recrystallized grains grow from the boundary of the original grain and gradually grow to replace the original grain.

**Figure 8:**
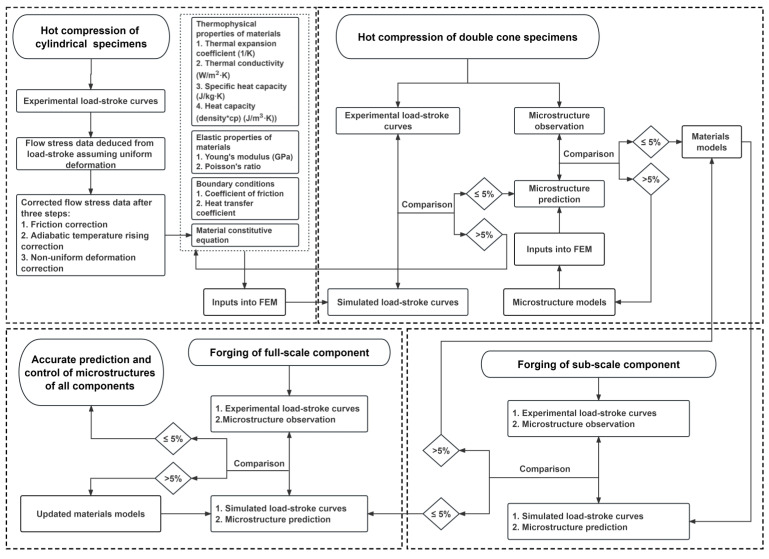
Workflow to predict microstructures of the full-scale component.

Figure 8 shows the workflow for predicting microstructures from cylindrical compression to DC compression and the forging of production-scale components.

Firstly, flow curves were obtained using cylindrical compression specimens over a range of temperatures, strains, and strain rates. From the measured load-stroke curves, the corresponding true stress-true strain data [4], i.e., flow curves, were obtained [5]. The flow curves were then corrected to account for the effects of friction [6], adiabatic temperature rise, and non-homogeneous deformation [7,8], such that the constitutive models were established to describe alloy behavior during hot deformation and were integrated into the finite element simulation.

Secondly, double-cone (DC) specimen compression tests were conducted to generate a wider range of strain and strain rates [9] within a single specimen [10]. The measured load-stroke and final dimensions from the DC tests were compared with the finite element simulation using the corrected flow curve data. When the difference between the predicted and experimental load-stroke data and the final dimensions was within an acceptable range, typically less than 5%, the flow curves and finite element forging simulation were considered validated.

Thirdly, the grain microstructures of the compressed cylindrical specimens were characterized to calibrate the grain size model. The predicted processing conditions, including strain, strain rate, and temperature, were inputs for the grain size model to quantify the effect of processing parameters on the grain microstructure inside the DC specimen.

Furthermore, selected locations within the deformed double cones were experimentally characterized for grain microstructures and used to validate the simulated grain size [11]. The selected locations have processing conditions that cover a range of thermo-mechanical conditions that are typically encountered in industrial forgings [12]. When the difference between the experimentally observed and the simulated grain size falls to an acceptable value, the grain size model is accepted.

This workflow is vital to establishing reliable prediction of grain microstructures from laboratory cylindrical compression testing to DC compression testing and production-scale component forging.

## 4. Conclusions

A three-step procedure is outlined to establish modeling and simulation for the prediction of grain microstructures in laboratory specimens. The constitutive model and flow curve data for a powder-metallurgy high-cobalt superalloy are calibrated with the flow curve data of cylindrical compression specimens and with friction, adiabatic heating, and non-homogeneous deformation corrections. The hot deformation behavior and simulation tool are validated with double-cone specimens, which have a much broader range of effective strain; thus, the location-specific processing conditions are reliably predicted. The grain microstructures of the cylindrical and DC specimens are experimentally characterized, and they are used to calibrate and validate grain microstructure models. The effects of thermomechanical processing on the grain microstructure in a high-cobalt nickel-base superalloy are predicted and comparable with the experimental observations. To predict the grain microstructure during thermomechanical processing, this integrated experimental modeling approach enables the calibration and validation of constitutive models and simulation with cylindrical and DC compression specimens and provides modeling and computational capabilities that are capable of industrial forging simulations.

## Data Availability

Data is contained within the article.
